# Causal relationship between immune cells and pulmonary arterial hypertension: Mendelian randomization analysis

**DOI:** 10.1097/MD.0000000000039670

**Published:** 2024-09-13

**Authors:** Dan Du, Jia-Yong Qiu, Jing Zhao, Ya-Dong Yuan

**Affiliations:** a Department of Respiratory and Critical Care Medicine, The Second Hospital of Hebei Medical University, Shijiazhuang, China.

**Keywords:** immunocyte cells, Mendelian randomization, observational study, pulmonary arterial hypertension

## Abstract

Immunity and inflammation in pulmonary arterial hypertension (PAH) has gained more attention. This research aimed to investigate the potential causal connections between 731 immunophenotypes and the likelihood of developing PAH. We obtained immunocyte data and PAH from openly accessible database and used Mendelian randomization (MR) analysis to evaluate the causal association between each immunophenotype and PAH. Various statistical methods were employed: the MR-Egger, weighted median, inverse variance weighted (IVW), simple mode, and weighted mode. In the study of 731 different types of immune cells, it was found that 9 showed a potential positive connection (IVW *P* < .05) with increased risk of PAH, while 19 had a possible negative link to decreased risk. Following false discovery rate (FDR) adjustment, the analysis using the IVW method demonstrated that 5 immune phenotypes were significantly associated with PAH (FDR < 0.05, OR > 1). Conversely, there was a negative correlation between PAH and 4 immune cell types (FDR < 0.05, OR < 1). Sensitivity analyses suggested the robustness of all MR findings. This research, for the first time, has revealed indicative evidence of a causal link between circulating immune cell phenotypes and PAH through genetic mechanisms. These results underscore the importance of immune cells in the pathogenesis of PAH.

## 1. Introduction

Pulmonary arterial hypertension (PAH) describes a cluster of significant cardiopulmonary and vascular disorders. It is mainly identified by elevated pressure in the pulmonary artery and heightened resistance in pulmonary vessels, ultimately leading to right heart failure.^[1]^ The intricate pathophysiology of PAH remains not completely comprehended. Nevertheless, there is a general consensus that vascular remodeling plays a critical role in the onset and advancement of PAH.^[2]^ Accumulating evidence indicates that inflammation and dysregulated immunity mutually influence maladaptive organ perfusion and congestion as key pathogenic drivers of vascular remodeling. The role of cytokines and immune cells in PAH gains increasing attention, providing novel mechanistic insights into the underlying immunopathology. Inflammatory mediators and cellular immune circuits link the local inflammatory landscape in the lung and heart as drivers of PAH.^[3]^ This process involves monocytes, macrophages, natural killer (NK) cells, dendritic cells (DCs), and T and B lymphocytes with distinct and organ-specific pro- and anti-inflammatory roles in homeostasis and disease.^[4]^

Despite the growing amount of research investigating the correlation between immune cells, the inflammation they produce, and PAH, the majority of these studies have been cross-sectional or observational. These studies have been unable to definitively prove a causal link between PAH and immune cells. Additionally, the limited sample sizes in these studies restrict the strength and scope of the findings. Thus, further exploration is necessary to determine the causal relationship between PAH and immune cells.

**Figure 1.**
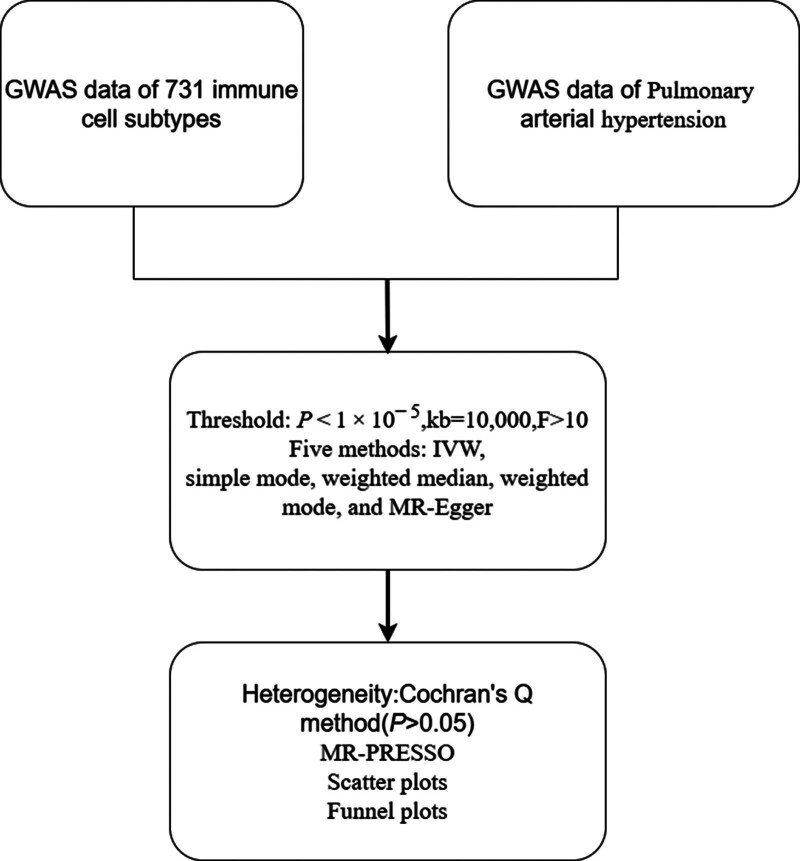
The protocol of our study procedure.

Utilizing genetic variations, Mendelian randomization is an epidemiological method that uses individual nucleotide polymorphisms as instrumental factors to replace target variables of exposure. This allows for the assessment of the causal relationship between exposure to a specific outcome.^[5]^ Therefore, the Mendelian randomization (MR) method was employed to investigate the causal link between immunocyte cells and the risk of PAH through genetic variation. Additionally, the study aimed to identify potential immunophenotypes linked to PAH, providing valuable insights for disease diagnosis and treatment. The study protocol is outlined in Figure 1.

## 2. Methods

### 2.1. Data extraction

**Figure 2.**
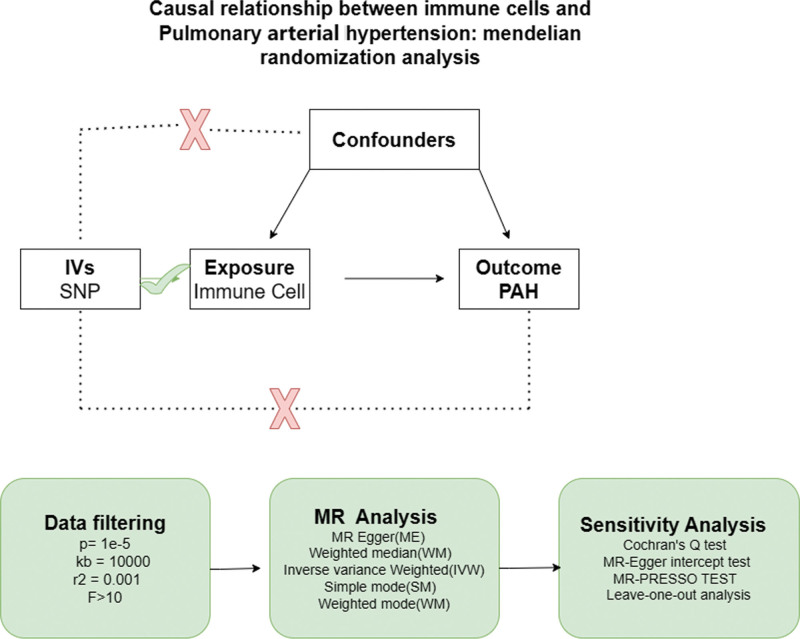
The hypothesis of the MR Analysis.

The immune cells used in the study were obtained from existing publications ranging in serial numbers from GCST90001391 to GCST90002121.^[6]^ A sum of 731 immune cells were ultimately incorporated in this analysis. Data for the outcome samples were extracted from the Genome-Wide Association Studies (GWAS) database, which included 125 cases and 162,837 controls of European populations (Table 1). Figure 2 illustrates the hypothesis. This investigation involved a reexamination of previously gathered and disclosed data, and as such, did not necessitate further ethical clearance.

**Table 1:** Details of the genome-wide association studies and datasets.

Exposure or outcome	Sample size	Ancestry	Links for data download
Immunophenotypes	731 immunological characteristics	Sardinian descent	http://ftp.ebi.ac.uk/pub/databases/gwas/summary statistics/ (GCST90001391–GCST90002121)
Pulmonary arterial hypertension	162,837 controls and 125 cases	European (Males and Females)	https://gwas.mrcieu.ac.uk/datasets/finn-b-I9_HYPTENSPUL/

### 2.2. Instrumental variables selection

Based on recent research findings, a significance threshold of 1 × 10^−5^ was determined for each immunological trait’s instrumental variables (IVs). The “TwoSampleMR” software package was utilized to guarantee the loci’s independence for our IVs. A threshold for linkage disequilibrium was established at *R*^2^ < 0.001, with an aggregation distance of 10,000 kb, using data from the European (EUR) subset of the 1000 Genomes Project.^[7]^

For each single nucleotide polymorphism (SNP), we meticulously gathered essential details such as the effect allele, the effect size shown as the β value, the standard error, and *P*-value. To assess the robustness of our instrumental variables, we determined the percentage of variance explained (*R*^2^) and the F statistic. The equations utilized were *R*^2^ = 2 × MAF × (1 − MAF) × β2 and F = *R*^2^ × (n − k − 1)/[k × (1 − *R*^2^)], where MAF represents the minor allele frequency of the SNP, “n” representing the sample size, and “k” indicating the quantity of IVs utilized.^[8,9]^

### 2.3. Statistical analysis and methodology

In our study, a variety of techniques were used to investigate possible causal connections between immunophenotypes and PAH. These approaches encompassed fixed/random-effects inverse variance weighting (IVW),^[10]^ the weighted median approach,^[11]^ MR-Egger regression,^[12]^ as well as both simple and weighted mode techniques.^[13]^

We selected the IVW approach as our main analytical technique due to its broad approval for delivering accurate effect estimates in MR investigations,^[14,15]^ After implementing the Benjamini–Hochberg False Discovery Rate (FDR) method for multiple testing adjustment of the IVW outcomes, immunophenotypes showing a FDR < 0.05 were considered to demonstrate a meaningful causal association with PAH.^[16]^

### 2.4. Statistical tools for assessing sensibility

To address potential heterogeneity among the selected SNPs, Cochran *Q* test and MR-Egger regression were implemented. If heterogeneity was detected (*P* *<* .05), the random-effects IVW approach was employed. Conversely, if no significant heterogeneity was found, the fixed-effects IVW method was utilized.^[10,12,17]^

Additionally, to further refine our analysis, the MR-PRESSO test was employed. This test is designed to identify and exclude potential outliers among SNPs that could significantly skew our results. It achieves this through a global heterogeneity test.^[18]^

In our analysis, to visually assess the influence of outliers and the robustness of our conclusions, we integrated scatterplots and funnel plots. Scatterplots were especially valuable in verifying that our outcomes were not disproportionately impacted by any outliers. Additionally, funnel plots offered a visual depiction of both the magnitude and uniformity of the relationships, reinforcing the lack of notable heterogeneity.

All statistical analyses were carried out with the R software packages: TwoSampleMR (version 0.5.6), Mendelian randomization,^[14]^ and MR-PRESSO (version 1.0) in R software version 4.2.2. Statistical significance was determined by a *P* value < .05.

## 3. Results

### 3.1. Forward instrumental variable

In this study, the GWAS data of 731 immunocyte phenotypes were screened for IVs, and all of the IVs had F-values >10, and there was no weak instrumental variable bias. See Files 1, Supplemental Digital Content, http://links.lww.com/MD/N549 which detailed outcome data for each SNP.

### 3.2. Causal effects of immunocyte on PAH

**Figure 3.**
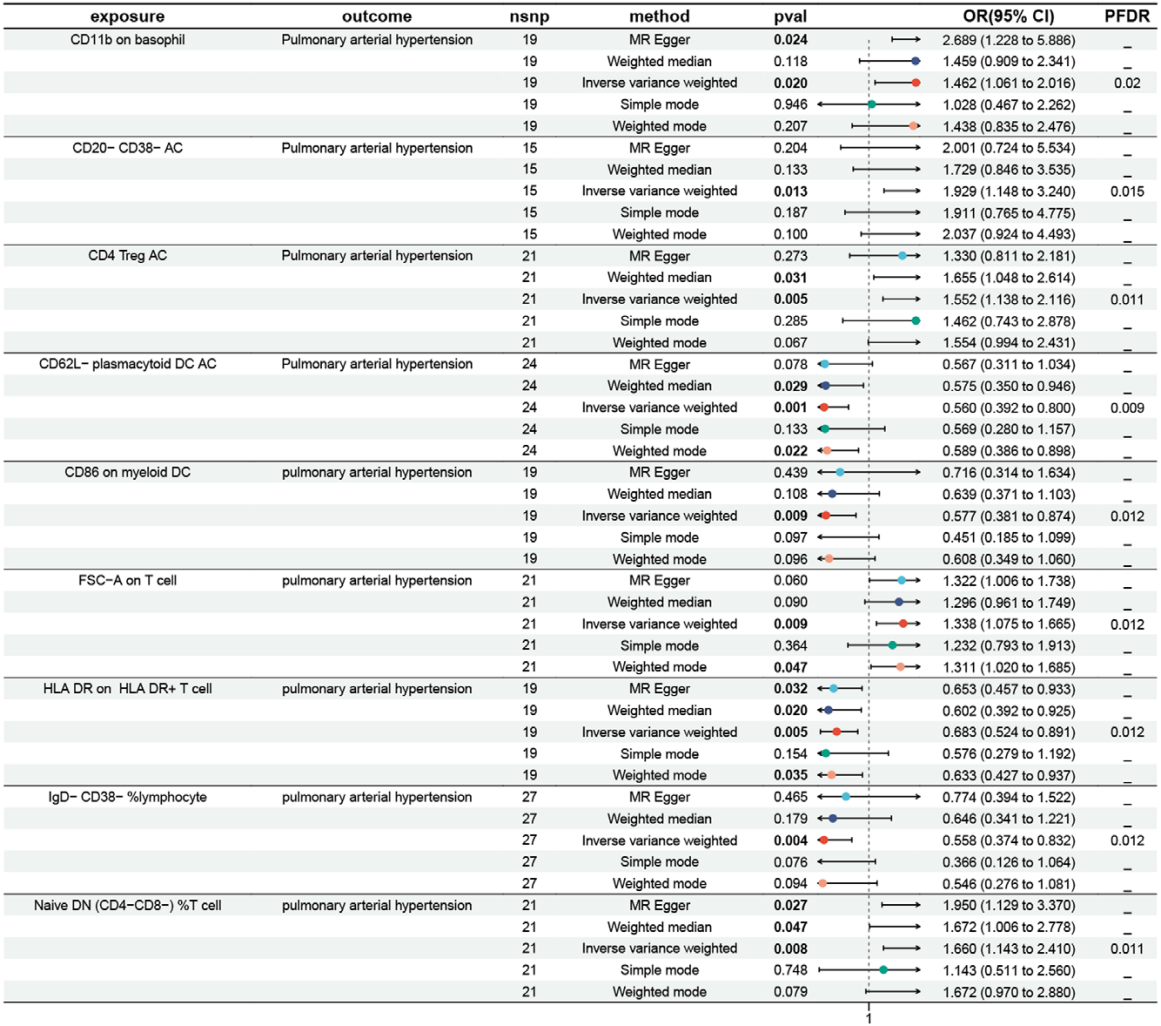
Causal effects of immunocyte on PAH.

We performed a two-sample MR analysis to investigate immunocyte on PAH. The false discovery rate (FDR) was controlled using the Benjamini–Hochberg method for *P*-value adjustment, with the IVW method serving as the primary analytical technique. We set a FDR threshold of 0.05, which led to the identification of 1 immune trait. The results of the genetically predicted IVW method for 2 groups of immune cells against PAH are shown in Figure 3. The MR analysis findings indicated a positive genetic causal link between PAH and 5 immune cells (OR > 1, *P* < .05), specifically involving CD11b on basophil, CD20-CD38-AC, CD4 Treg AC, FSC-A on T cell, Native DN(CD4-CD8-)%T cell. Additionally, 4 immune traits were found to reduce the risk of PAH (*P* < .05, OR < 1). Notably, we observed an inverse correlation between PAH risk and the following immune cells: CD86 on myeloid DC, HLA DR on HLA DR + T cell, IgD-CD38-%lymphocyte, CD62L-plasmacytoid DC AC. A FDR of 0.05 was set, leading to the identification of 1 immune trait. The outcomes of MR analysis revealed a positive genetic causal relationship between PAH and 5 immune cells(OR > 1, *P* < .05), such as CD11b on basophil, CD20-CD38-AC, CD4 Treg AC, FSC-A on T cell, Native DN(CD4-CD8-)%T cell, remaining 4 traits reduces the incidence of PAH (*P* < .05, OR < 1), It was noted that the following immune cells exhibited an inversely connection with PAH risk: CD86 on myeloid DC, HLA DR on HLA DR + T cell, IgD-CD38-%lymphocyte, CD62L-plasmacytoid DC AC. See Files 2, Supplemental Digital Content, http://links.lww.com/MD/N549 which detailed the 5 methods of MR analysis and see Figures S1 and S2, Supplemental Digital Content, http://links.lww.com/MD/N548 which demonstrates scatter plots for 9 data items.

### 3.3. Forward sensitivity analysis

The results of the test of heterogeneity and the test of horizontal pleiotropy showed that *P* > .05, so it can be concluded that there is no heterogeneity and horizontal pleiotropy among the IVs in this study. In addition, we performed MR-PRESSO global tests, suggesting that IVW results are reliable. Finally, the results of IVW analysis were used as the results of MR analysis and fixed effect model was used to show the final results (Table 2). Finally, see Figure S3 and S4, Supplemental Digital Content, http://links.lww.com/MD/N548 which the funnel plot displayed no evidence of asymmetry, indicating the absence of directional horizontal pleiotropy.

**Table 2:** Mendelian randomization analysis and sensitivity analysis of immune cells associated with PAH risk.

Panel	Immunocyte		MR analysis		Heterogeneity		Pleiotropy		AT global outlier test	
	Exposure	nSNPs	OR (95% CI)	PFDR	*Q*	*P*-value	Intercept	*P*-value	RSSOBs	*P*-value
TBNA	HLA DR on HLA DR + T cell	19	0.68 (0.52–0.89)	0.005	18	.783	4.052	.812	16.406	.753
TBNA	FSC-A on T cell	21	1.34 (1.07–1.66)	0.009	20	.851	8.425	.559	14.315	.895
B cell	IgD- CD38- %lymphocyte	27	0.56 (0.37–0.83)	0.004	26	.231	6.671	.614	33.244	.267
Maturation stages of T cell	Naive DN (CD4-CD8-) %T cell	21	1.66 (1.14–2.41)	0.008	17	.622	12.325	.425	35.160	.141
B cell	CD20-CD38-AC	15	1.93 (1.15–3.24)	0.013	14	.714	8.425	.559	11.681	.795
cDc	CD86 on myeloid DC	19	0.58 (0.38–0.87)	0.010	18	.276	6.671	.614	22.567	.345
Myeloid cell	CD11b on basophil	19	1.46 (1.06–2.02)	0.020	18	.736	8.000	.499	15.517	.749
cDc	CD62L-plasmcytoid DC AC	24	0.56 (0.39–0.80)	0.019	23	.257	−0.004	.959	27.977	.338
Treg	CD4 Treg AC	21	1.55 (1.14–2.12)	0.006	20	.490	12.325	.425	20.522	.587

### 3.4. Reverse instrumental variable

In this study, the GWAS data on PAH were screened for IVs, and all IVs had F-values >10 without weak instrumental variable bias. See Files 3, Supplemental Digital Content, http://links.lww.com/MD/N549 which detailed outcome data for each SNP.

### 3.5. Causal effects of PAH on immunocyte

**Figure 4.**
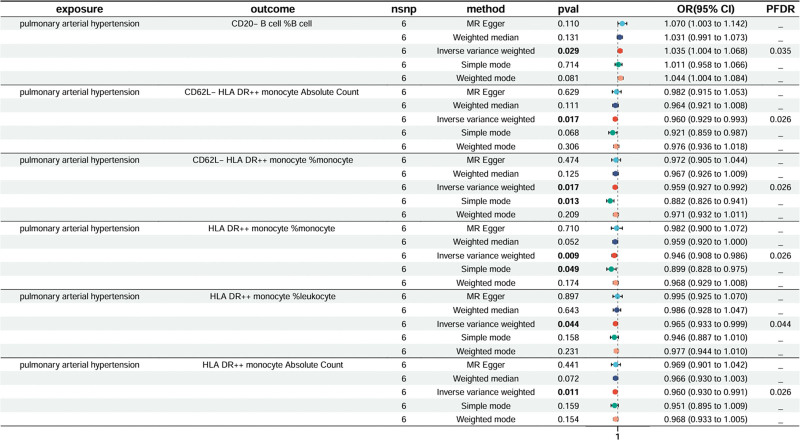
Causal effects of PAH on immunocyte.

We applied the same approach to investigate the impact of PAH on immune traits. A FDR of 0.05 was set, resulting in the observation of 6 immune trait. The results of the genetically predicted IVW method for 2 groups of PAH against immune cells are shown in Figure 4. Treg panel: CD20-%B cell, CD62L-HLA DR++monocyte AC, CD62L-HLA DR++ monocyte %monocyte, HLA DR++ monocyte %monocyte, HLA DR++ monocyte Absolute Count, HLA DR++ monocyte %leukocyte. See Files 4, Supplemental Digital Content, http://links.lww.com/MD/N549 which detailed the 5 methods of MR analysis.

### 3.6. Reverse sensitivity analysis

The results of the test of heterogeneity and the test of horizontal pleiotropy showed that *P* > .05, so it can be concluded that there is no heterogeneity and horizontal pleiotropy among the IVs in this study, we performed MR-PRESSO global tests, suggesting that IVW results are reliable. Finally, the results of IVW analysis were used as the results of MR analysis and fixed effect model was used to show the final results (Table 3).

**Table 3:** Mendelian randomization analysis and sensitivity analysis of PAH on Immunocyte.

Panel	Immunocyte		MR analysis		Heterogeneity		Pleiotropy		AT global outlier test	
	Exposure	nSNPs	OR (95% CI)	PFDR	Q	*P*-value	Intercept	*P*-value	RSSOBs	*P*-value
B cell	CD20-B cell %B cell	6	1.04 (1.00–1.07)	0.035	5	.899	−0.052	.318	4.052	.812
cDC	CD62L-HLA DR++ monocyte AC	6	0.96 (0.93–0.99)	0.026	5	.517	−0.034	.520	8.425	.559
cDC	CD62L-HLA DR++ monocyte %monocyte	6	0.96 (0.93–0.99)	0.026	5	.563	−0.020	.706	6.671	.614
TBNK	HLA DR++ monocyte %monocyte	6	0.95 (0.91–0.99)	0.026	5	.131	−0.058	.395	18.618	.275
	HLA DR++ monocyte Absolute Count	6	0.96 (0.93–0.99)	0.026	5	.410	−0.014	.796	8.000	.499
TBNK	HLA DR++ monocyte %leukocyte	6	0.97 (0.93–1.00)	0.044	5	.330	−0.047	.407	12.325	.425

## 4. Discussion

In this study, we investigated the causal relationship between 731 different immune phenotypes and PAH using bidirectional MR analysis, our findings indicated some suggestive evidence for a causal link between 9 types of immune cells, including B cells, cDC, T cells, mature T cells, myeloid cells, and TBNK (T cells, B cells, natural killer cells), in the context of forward MR and PAH. In the reverse MR analysis, 6 immune cell types (cDC, B cells, and TBNK) showed a causal connection with PAH. Importantly, no bidirectional causal relationship was observed for the same immune cell phenotype.

Our findings indicated that there is a positive correlation between 5 different types of immunocytes and the likelihood of developing PAH. Of these, CD4 Treg AC has not yet been directly investigated in relation to PAH. Nonetheless, other studies have shown an increase in Treg levels in the peripheral blood of PAH patients.^[19]^ This could be due to immune biomarkers, with the ratio of regulatory T cells within CD4 (+) T cells being notably higher in the PAH group compared to the non-PAH group.^[20]^ Additionally, as per research by Tomohiko Ishibash and colleagues, the IL-6/gp130 signal within CD4 + T cells plays a crucial role in the development of PAH. It was found that certain regulatory T cells with a CD4 phenotype actually promote PAH.^[21]^ The presence of myeloid inhibitory cells marked by CD11b in the peripheral blood of PAH patients was significantly elevated and implicated in the process of vascular remodeling,^[22]^ aligning with the findings of this study. B lymphocytes are known to induce apoptosis of pulmonary artery endothelial cells, trigger an inflammatory response, and exacerbate pulmonary vascular remodeling by releasing a variety of autoantibodies. Some evidence suggests that abnormalities in the B cell immune response in individuals with idiopathic PAH and connective tissue disease-associated PAH may have implications for survival rates in those with hereditary and idiopathic PAH.^[23,24]^ However, the precise role of B cells is not yet fully understood. It is unclear whether B cells directly contribute to endothelial cell damage, amplify vascular inflammation, or suppress vascular inflammation. These questions have not been definitively answered and warrant further investigation.^[23]^ Research has shown that a CD20-targeted monoclonal antibody can effectively decrease the release of circulating PAH antibodies and inflammatory factors. Clinical trials testing the efficacy of CD20 monoclonal antibody in treating systemic sclerosis-associated PAH are ongoing, suggesting that CD20-marked B cells play a role in pulmonary vascular remodeling.^[25]^ This study found a positive correlation between CD20-CD38-AC and PAH.

The analysis using MR also revealed an inverse relationship between the 6 immunocyte phenotype and PAH, suggesting potential implications for upcoming PAH therapies. Dendritic cells (DC), the antigen-presenting cells, are involved in the stimulation and propagation of primary T cells, while mature DC demonstrates proficient recognition and presentation of antigens. Consequently, there is a reduction in circulating conventional DC (cDC) in IPAH individuals, indicating a beneficial impact of cDC.^[26]^ This research aligns with previous studies in the literature. Moreover, the link between NK cells and PAH is particularly significant. Edwards et al noted a decrease in NK cell activity in PAH patients, which was associated with disease progression and a higher mortality risk, especially in patients with CTD-PAH. These findings suggest that NK cells may have an inhibitory role in PAH, indicating their potential as a target for therapy.^[27]^ At present, the study of immune cell phenotype in PAH is still lacking. Further investigations are necessary to explore the impact of immunophenotypes in PAH.

During our investigation into the inverse relationship between 731 types of immune cells and PAH, we found that the immune cell types with causal connections did not show inverse causal links. Put simply, certain immune cell types affecting the advancement or relief of pulmonary hypertension do not have a mutual effect on the formation of PAH.

This research utilized MR Analysis for the first time to explore the causal link between immune cells and pulmonary hypertension. By utilizing MR Analysis, the study was able to effectively address confounding variables and minimize the impact of ethical considerations, enhancing the credibility of the results compared to conventional epidemiological statistical methods. Furthermore, the study utilized the most recent GWAS data available with the largest sample size in the database, further bolstering the reliability of the findings. A total of 731 immune cell types were examined in the study, with a thorough bidirectional validation process conducted to enhance the robustness of the conclusions and offer valuable insights for future research endeavors. There are limitations to the study. Initially, as the immune cell samples mainly came from individuals of Sardinian descent, and PAH cases were solely from European populations, there could be potential selection bias. Despite using MR Analysis to randomize study subjects, the small sample size of immune cells requires consideration of bias related to age, gender, and ethnicity. Additionally, while strict regulations were followed for sample screening and injections in this study to improve result reliability, the lack of basic research to support these findings requires further investigation into functionality and underlying mechanisms. Therefore, after meeting necessary conditions, subgroup analyses based on variables such as age, ethnicity, and gender can be conducted to gather more data and improve result generalizability.

## 5. Conclusion

Our study focused on exploring the immune-related pathways involved in pulmonary hypertension by examining various cell subtypes. By analyzing data from GWAS, we have identified specific immunophenotypes that are associated with pulmonary hypertension. This methodology assists in identifying potential protein targets for drug development and lays the foundation for future therapeutic approaches that target the immune system.

## Author contributions

**Conceptualization:** Dan Du, Jia-Yong Qiu, Jing Zhao.

**Data curation:** Dan Du.

**Formal analysis:** Dan Du.

**Methodology:** Dan Du.

**Software:** Jia-Yong Qiu.

**Supervision:** Ya-Dong Yuan.

**Writing – original draft:** Dan Du.

## Supplementary Material


