# Supplementary material for: Causal relationship between immune cells and pulmonary arterial hypertension: Mendelian randomization analysis
Source: Medicine (Baltimore). 2024 Sep 13;103(37):e39670. doi: 10.1097/MD.0000000000039670 (PMC11404942; doi:10.1097/MD.0000000000039670)
Supplement: Supplementary file 2 [file medi-103-e39670-s002.pdf]

Supplement figure 1. The scatter plot of Immunocyte on PAH in positively effect

Supplement figure 2. The scatter plot of Immunocyte on PAH in negatively effect

Supplement figure 3. The funnel plot of Immunocyte on PAH in positively effect

Supplement figure 4. The funnel plot of Immunocyte on PAH in negatively effect

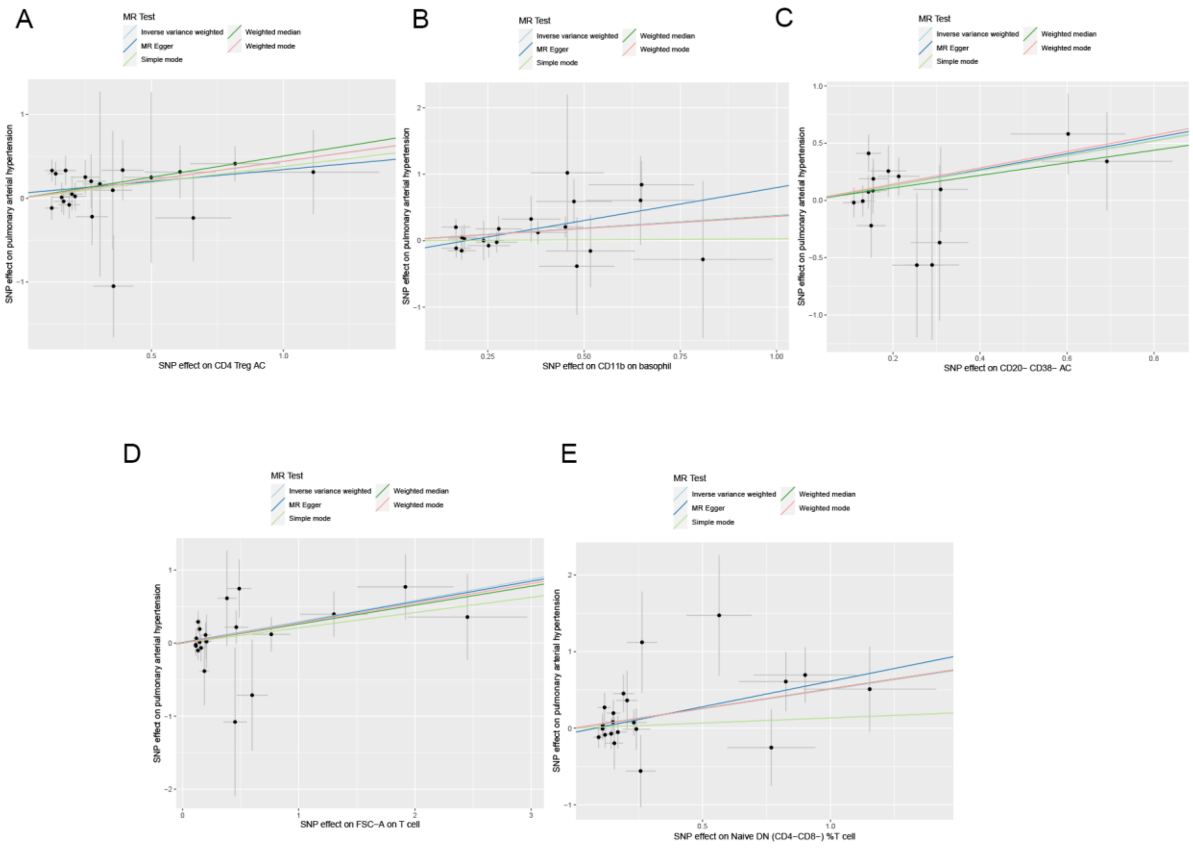

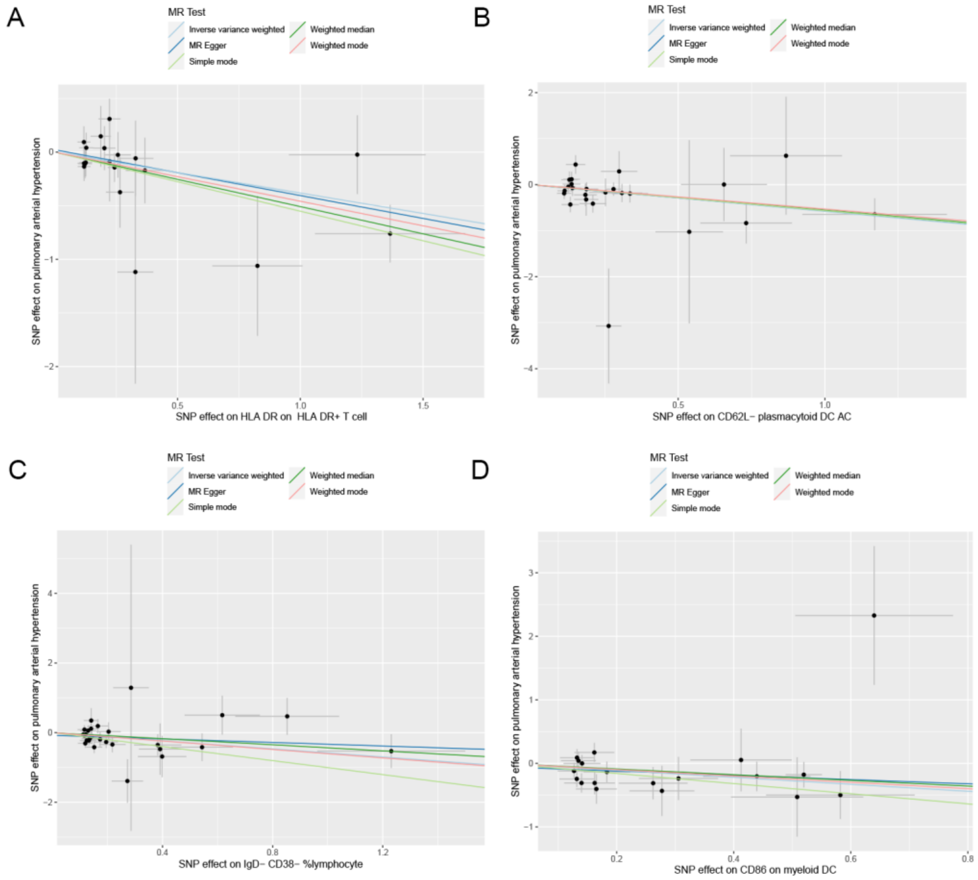
A
MR Test
Inverse variance weighted
MR Egger
Simple mode
Weighted median
Weighted mode
SNP effect on pulmonary arterial hypertension
SNP effect on HLA DR on HLA DR+ T cell
B
SNP effect on CD62L− plasmacytoid DC AC
C
SNP effect on IgD− CD38− %lymphocyte
D
SNP effect on CD86 on myeloid DC

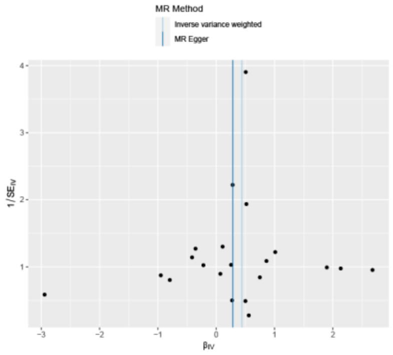

CD4 Treg AC

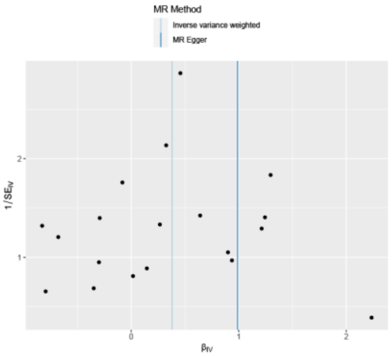

CD11b on basophil

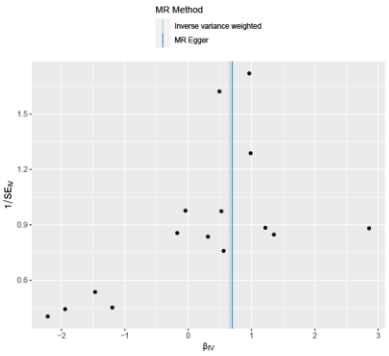

CD20-CD38-AC

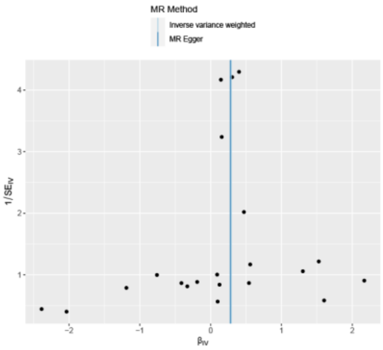

FSC-A on T cell

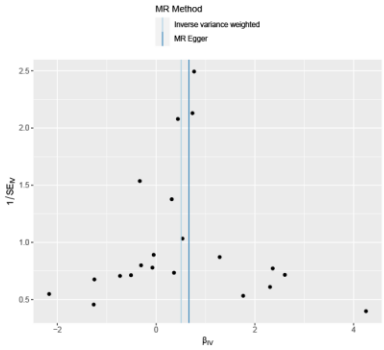

Naive DN(CD4-CD8)%T cell

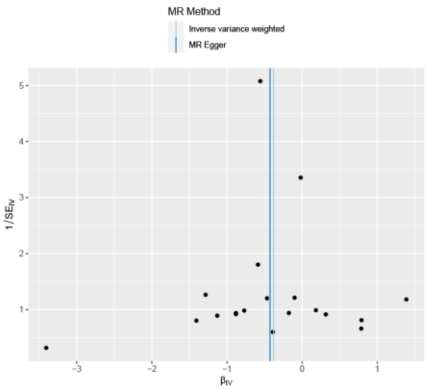

HLA DR on HLA DR+ T cell

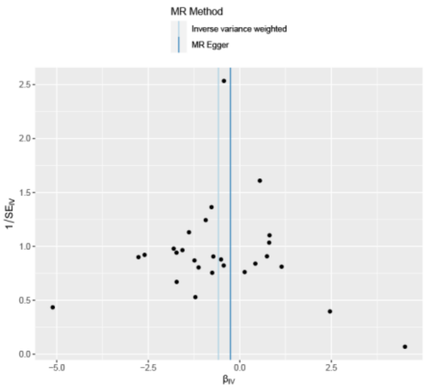

IgD-CD38-%lymphocyte

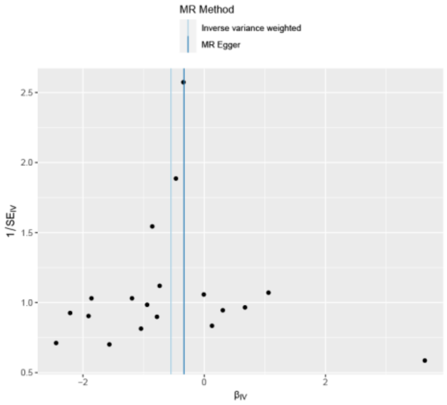

CD86 on myeloid DC

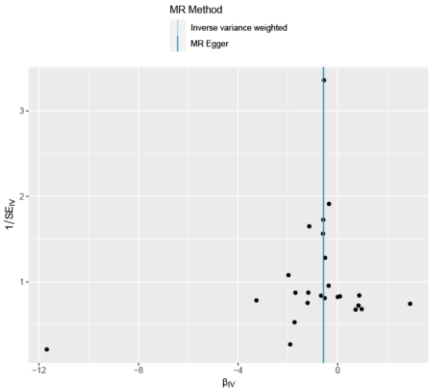

CD62-plasmacytoid DC AC
